# Gut neurotoxin p-cresol induces brain-derived neurotrophic factor secretion and increases the expression of neurofilament subunits in PC-12 cells

**DOI:** 10.3934/Neuroscience.2022002

**Published:** 2021-12-23

**Authors:** Gigi Tevzadze, Tamar Barbakadze, Elisabed Kvergelidze, Elene Zhuravliova, Lali Shanshiashvili, David Mikeladze

**Affiliations:** 1 4-D Research Institute, Ilia State University, 3/5 Cholokashvili av, Tbilisi, 0162, Georgia; 2 School of Natural Sciences and Medicine, Institute of Chemical Biology, Ilia State University, 3/5 Cholokashvili ave, Tbilisi, 0162, Georgia; 3 I. Beritashvili Center of Experimental Biomedicine 14, Gotua Str., Tbilisi 0160, Georgia

**Keywords:** p-cresol, microbiome, BDNF, oxytocin, neurofilament

## Abstract

Increased p-cresol levels reportedly alter brain dopamine metabolism and exacerbate neurological disorders in experimental animals. In contrast to toxic concentrations, low doses of p-cresol may have distinct effects on neuronal metabolism. However, the role of p-cresol in synapse remodeling, neurite outgrowth, and other anabolic processes in neurons remains elusive. We propose that low doses of p-cresol affect neuronal cell structural remodeling compared with the high concentration-mediated harmful effects. Thus, the effects of p-cresol on the secretion of brain-derived neurotrophic factor (BDNF) and neurofilament subunit expression were examined using rat pheochromocytoma cells (PC-12 cells). We observed that low doses of p-cresol potentiated nerve growth factor-induced differentiation via secretion of BDNF in cultured PC-12 cells. Opioidergic compounds modulated these p-cresol effects, which were reversed by oxytocin. We propose that this effect of p-cresol has an adaptive and compensatory character and can be attributed to the induction of oxidative stress. Accordingly, we hypothesize that low doses of p-cresol induce mild oxidative stress, stimulating BDNF release by activating redox-sensitive genes. Given that the intestinal microbiome is the primary source of endogenous p-cresol, the balance between gut microbiome strains (especially Clostridium species) and opioidergic compounds may directly influence neuroplasticity.

## 1. Introduction

It is well-known that the gut microbiota modulates behavior and contributes to neurological disorders [1],[2]. Dysbiotic gut microbiota could contribute to the development of autism spectrum disorders (ASD), epilepsy, and anxiety [3],[4]; however, the underlying mechanisms that induce these disorders remain unknown. *Clostridium difficile* is known to play a critical role in the development of emotional and cognitive complications in ASD, epilepsy, and anxiety [3],[5],[6]. p-Cresol is the most active neurotoxic substance produced by Clostridium [7]. This toxin can cross the blood-brain barrier and, by altering dopamine metabolism, changes dopamine receptor activity in different brain areas [8],[9]. Recently, we have reported that p-cresol can induce differential expression of GluN2B and GluN2A subunits of the N-methyl-d-aspartate receptor (NMDAR) in the hippocampus and nucleus accumbens of healthy and audiogenic seizure-prone rats [10],[11]. Given that increased p-cresol levels alter brain dopamine metabolism and exacerbate neurological disorders in experimental animals [6], we propose that the differences in the clinical manifestations in patients with certain neurological disorders may be attributed to the intensity of Clostridium bacterial colonization of the intestinal tract, and correspondingly to the amount of p-cresol in the brain.

In contrast to toxic concentrations, specific doses of p-cresol may have distinct effects on neuronal metabolism. However, the role of p-cresol in synapse remodeling, neurite outgrowth, and other anabolic processes in neurons needs to be clarified. Accordingly, we investigated the effects of p-cresol on the secretion of brain-derived neurotrophic factor (BDNF) and neurofilament (NF) subunit expression in model experiments using rat pheochromocytoma cells (PC-12 cells). Cultured PC-12 cells display a chromaffin cell-like morphology but undergo rapid changes following treatment with nerve growth factor (NGF), including neurite outgrowth. NGF is known to induce neurite development and increase the levels of NF proteins in PC-12 cells. [12]. These cells are widely employed as model systems to examine the signal transduction process (especially dopaminergic) in neurons [13]. In the present study, we observed that low doses of p-cresol could potentiate NGF-induced differentiation by secreting BDNF in cultured PC-12. In addition, we noted that opioidergic compounds modulated these effects of p-cresol.

## 2. Materials and methods

### 2.1. Reagents

Pheochromocytoma cells (PC-12, ATCC® CRL-1721™) were purchased from ATCC. p-Cresol was purchased from UHN Shanghai R&D Co. Ltd. Anti-NF-M/H (RMdO-20) primary antibody (sc-32273), poly-D-lysine (sc-136156), and RIPA lysis buffer system (sc-24948) were purchased from Santa Cruz Biotechnology. Horseradish peroxidase (HRP)-conjugated anti-mouse secondary antibody (ab6814) and a human BDNF ELISA Kit (ab99978) were purchased from Abcam plc. Native mouse NGF 2.5S protein (99%) (N-245) was purchased from Alomone Labs. Dulbecco's modified Eagle's medium (DMEM), high glucose, GlutaMAX™ Supplement, pyruvate (#10569010, Gibco), horse serum (HS), fetal bovine serum (FBS), and micro BCA™ Protein Assay Kit (cat.no: 23235) were purchased from ThermoFisher Scientific. Albumin V fraction (Art. No. 8076.3; Roth), rizatriptan (Rizeptam 10 mg; Laboratirios Lesvi, Spain), and enkephalin M (Sigma-Aldrich; M6638) were also procured.

### 2.2. PC12 cell cultures

PC-12 cells were cultured in T25 flasks (Greiner Bio-One GmbH, cat. No. 690 170) in a humidified atmosphere containing 5% CO_2_ at 37 °C in high-glucose DMEM, supplemented with 10% heat-inactivated HS (Sigma-Aldrich, cat no. H1138), 5% FBS (Sigma-Aldrich, cat. No. F2442), 100 U/mL penicillin, and 50 µg/mL gentamicin sulfate. To induce differentiation, PC-12 cells were incubated in low serum-containing DMEM (1% HS and 1% FBS), supplemented with 100 ng/mL NGF for 5 days. The NGF-containing medium was replaced every other day. The cells were scored as differentiated if the neurites were longer than twice the diameter of one cell body. Cell viability was measured by staining cells with trypan blue dye (Bio-Rad, cat no. 145-0013) and using an automated cell counter (TC 20TM; Bio-Rad, USA) to count live cells. Average neurite length was measured using Image J software (https://imagej.net/ImageJ).

### 2.3. Study design

Study design #1. PC-12 cells (2 × 10^6^ cells per sample) were pretreated with p-cresol (final concentration, 1 µM) for 5 days. Then, p-cresol addition was stopped, and NGF-containing medium was added for the next 5 days. Finally, the BDNF content was measured in medium (on day 5 after pre-incubation with p-cresol and day 10 after treatment with NGF and p-cresol), and PC-12 cells were used to analyze NFs. Cells were washed twice with cold PBS buffer, removed from the cell culture flasks using 0.025% trypsin–phosphate-buffered saline (PBS) buffer (incubation for 1 min), scraped (trypsin inactivation performed using aprotinin-containing PBS [1 mg/mL], followed by resuspension in PBS), and pelleted by centrifugation at 300 × *g*. Finally, the incubated PC-12 cells were solubilized in RIPA lysis buffer and passed through a 25-G needle 10 times using a 1 mL syringe. The total protein values were determined in cell lysates using micro BCA™ Protein Assay Kit. After cell lysis, nuclei and intact cells were sedimented at 720 × *g* for 5 min, and the supernatant was subjected to electrophoresis and western blotting.

Study design #2. PC-12 cells (2 × 10^6^ cells per sample) were treated with p-cresol (final concentration 1 µM) and/or rizatriptan (final concentration 1 µM) and/or enkephalin and/or oxytocin (final concentration 1 µM) for 5 days. BDNF was measured in the cell culture medium after incubation. Cell density was one million cells per ml. Total protein content in cell lysates was measured using micro BCA™ Protein Assay Kit.

### 2.4. Sodium dodecyl sulfate-polyacrylamide gel electrophoresis (SDS-PAGE) and western blotting

Proteins were separated on 8% SDS-polyacrylamide gels and transferred onto nitrocellulose membranes. Then, transfer and equal sample loading were confirmed by staining with Ponceau-S. Next, the nitrocellulose membrane was blocked with 5% bovine serum albumin in Tris-buffer saline/0.1% Tween 20 (TBS-T). The primary antibody was then incubated with the primary antibody diluted in 3% bovine serum albumin in 0.1%TBS-T for 1 h at room temperature. Next, the nitrocellulose membrane was rinsed with TBS-T and incubated for 1 h at room temperature in HRP-conjugated anti-mouse secondary antibody diluted in 3% bovine serum albumin in 0.1%TBS-T. After washing with TBS-T, the immune complexes were visualized using the enhanced chemiluminescence (ECL) method (GE Healthcare, Piscataway, NJ, USA). The band intensities in control and different samples were analyzed using ImageJ software (National Institutes of Health, Bethesda, MD).

### 2.5. BDNF assay

The BDNF content was measured in the medium using the BDNF Human BDNF ELISA Kit, according to the manufacturer's protocol. Briefly, 100–100 µL of the medium was added to wells and incubated overnight at 4 °C. Next, the wells were washed, and a biotinylated anti-BDNF antibody was added. After washing away the unbound biotinylated antibody, HRP-conjugated streptavidin was pipetted into wells. Then, the wells were washed, TMB (3,3′,5,5′-tetramethylbenzidine) substrate solution was added to wells, and the color developed in proportion to the amount of bound BDNF. After adding the stop solution, the color intensity was measured at 450 nm using an ELx808 microplate spectrophotometer (BioTek). Protein concentrations in each sample were measured using a BCA™ Protein Assay Kit and values of BDNF were corrected for the total amount of protein in the cell lysate of the samples.

### 2.6. Statistical analysis

For each experiment, data were obtained from at least three independent culture preparations. The results are expressed as the mean ± standard error of the mean. Statistical analyses were performed using one-way analysis of variance. The results were considered significant at *p < 0.05 and compared with the control group in a low serum medium (or NGF control group), and #p < 0.05 compared with p-cresol group.

## 3. Results

**Figure 1.**
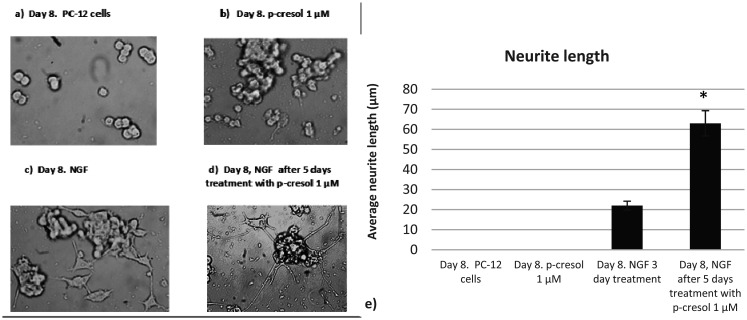
Microscope images of PC-12 cell culture (Jenco, Inverted light microscope; total magnification 400×): a) PC-12 cells without treatment at day 8; b) PC-12 cells treated with 1 µM p-cresol at day 8; c) PC-12 cells treated with NGF for 3 days, no pretreatment with 1 µM p-cresol; d) PC-12 cells treated with NGF for 3 days after 5-day pretreatment with 1 µM p-cresol. NGF, nerve growth factor. e) Quantification of the neurite length (µm) using Image J software (https://imagej.net/ImageJ). The values represent averages SEM from three independent cultures. The significance level was set at *p < 0.05, versus the NGF treated PC-12 cells.

Initially, neurite outgrowth in the presence of p-cresol was verified by microscopy, and found that 1 µM p-cresol pretreatment significantly increased neurite outgrowth (Figure 1).

**Figure 2.**
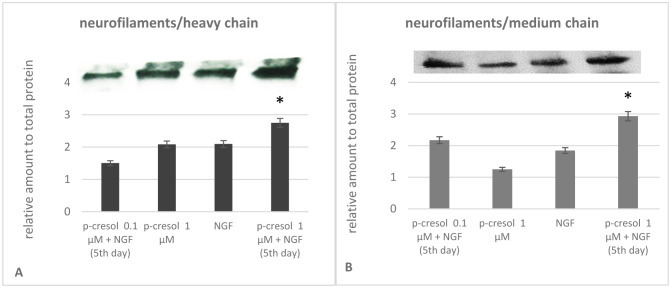
Analysis of the neurofilament (NF) heavy (H) and medium (M)-chain content in PC-12 cells. The cell lysates were loaded onto each well for immunoblotting, resolved by sodium dodecyl sulfate-polyacrylamide gel electrophoresis (SDS-PAGE), transferred to nitrocellulose membranes, and probed with NF-H/M antibodies. Bands from three experiments (n = 3) were quantified using Image J software (https://imagej.net/ImageJ). and plotted as a bar graph. Quantitative values represent mean normalized to total protein. The significance level was set at *p < 0.05, versus the NGF treated PC-12 cells. Data are derived from three independent experiments and are expressed as the relative change from the mean ± standard error of triplicate experiments' mean (SEM).

To determine the neurite outgrowth-promoting activity in the presence of p-cresol, we first examined the expression of NF subunits under the action of p-cresol. We observed that the expression of the NF-H chain was not significantly altered in the presence of NGF or 0.1µM p-cresol alone. In contrast, the addition of NGF after pretreatment with p-cresol significantly increased the NF content. Comparable results were obtained on analyzing medium chains. Protein expression was increased only following the addition of NGF in samples pretreated with 1 µM p-cresol and NGF (Figure 2). These data suggest that p-cresol increased the expression of NF subunits, confirming increased PC-12 differentiation in the presence of NGF.

**Figure 3.**
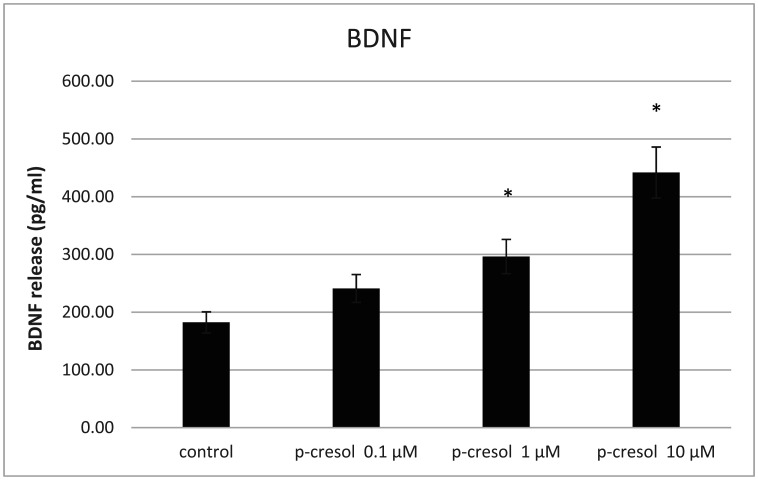
Analysis of the BDNF content in the incubation medium. The incubation period—24 hours. The significance level was set at *p < 0.05, displays versus the untreated PC-12 cells (control). Data are derived from three independent experiments (n = 3) and are expressed as the relative change from the mean ± standard error of triplicate experiments' mean (SEM). BDNF, brain-derived neurotrophic factor.

BDNF is involved in synaptogenesis and mediates neuronal cell survival. Oxidative stress can stimulate BDNF release from PC-12 cells via an autocrine or paracrine loop, requiring dopamine receptor activation [14]. Thus, low levels of oxidative stress can activate neurotropic factor-mediated autocrine/paracrine cell protective mechanisms [15]. Considering that p-cresol affects reactive oxygen species (ROS) generation [16], we propose that ROS accumulation via BDNF synthesis could positively affect adaptation to p-cresol-induced oxidative stress. Our results showed that low doses of p-cresol increased BDNF secretion (Figure 3).

**Figure 4.**
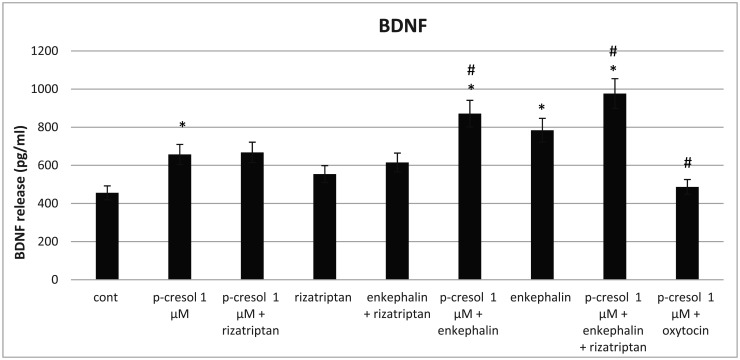
Analysis of the BDNF content in the incubation medium. The incubation period—48 hours. The significance level was set at *p < 0.05, versus the untreated PC-12 cells (control), and #p < 0.05, versus p-cresol 1 µM treated group. Data are derived from three independent experiments (n = 3) and are expressed as the relative change from the mean ± standard error of triplicate experiments' mean (SEM).

To determine the types of agonists that can modulate the effects of p-cresol, we introduced rizatriptan (a 5-HT1 serotonin receptor agonist), enkephalin (delta and mu-opioid receptor agonist), or oxytocin in the medium. Our results showed that the addition of enkephalin or enkephalin with rizatriptan after pretreatment with p-cresol increased BDNF secretion more than that induced by enkephalin and rizatriptan separately. (Figure 4). These data support the observation that the delta-opioid agonists may exert their neuroprotective role against oxidative stress via the BDNF-TrkB pathway [17]. In addition, we noted that oxytocin reversed the effects of p-cresol, suggesting that oxytocin receptors could decrease BDNF secretion.

## 4. Discussion

Plasma p-cresol levels in patients with uremia range between 100 and 250 µM [18]. At concentrations above 100 µM, p-cresol increases ROS generation induces cell cycle arrest and enhances cytotoxicity, as well as inflammation/atherosclerosis-related modulator production in endothelial and mononuclear cells [16]. Mice treated with these concentrations of p-cresol reportedly exhibit social interaction deficits associated with reduced excitability of dopaminergic neurons in the ventral tegmental area [6]. These neurons are known to project to the nucleus accumbens medial shell as part of a “socially engaged reward circuit” [19]. Accordingly, these data suggest that dopaminergic neurons are primary targets of p-cresol in the brain, which are known to be more susceptible to dopamine-induced oxidative stress.

Accumulated evidence suggests that toxic dopamine metabolites and ROS contribute to progressive neurodegeneration, as detected in Parkinson's disease [20]. Dopamine is metabolized via monoamine oxidase to 3,4-dihydroxyphenylacetaldehyde (DOPAL), a highly reactive aldehyde species, and H_2_O_2_ [21]. Thus, excessive cytosolic or extraneuronal dopamine induces marked dopamine oxidation to quinone species and ROS production. Moreover, dopamine beta-hydroxylase, an enzyme that catalyzes the conversion of dopamine to norepinephrine, is inactivated by p-cresol [8],[22]. Accordingly, inhibition of this enzyme by p-cresol can cause dopamine accumulation and enhance its catabolism via the formation of ROS and other toxic metabolites. This suggestion was confirmed by experiments showing that p-cresol increases brain dopamine metabolism and exacerbate autism-like behaviors in mice [4],[7]. Additionally, it is interesting to note that BDNF secretion from PC12 cells in response to oxidative stress requires autocrine dopamine signaling [14].

NFs are a class of neuron-specific intermediate filament heteropolymers that are composed of three subunit proteins (NF-light [NF-L], NF-medium [NF-M], and NF-heavy [NF-H]), based on their respective molecular masses [23]. NF subunits are integral components of synapses, and their elimination disrupts synaptic plasticity and impairs social memory [24]. NF is a core synaptic scaffold component that interacts with the GluN1 subunit of NMDAR [25] and the D1-dopamine receptor [26]. The formation of heterocomplexes between NF subunits and NR1 and D1R may be critical for the proper functioning of these receptors, including agonist-induced internalization and regulation of sensitivity toward agonists. Our results have shown that a low dose of p-cresol increases the expression of NF subunits.

Compared with the harmful effects of high-dose p-cresol, our findings revealed that low doses of p-cresol can impact neuronal cell structural remodeling. This protective effect is adaptive and compensatory and can be attributed to the induction of oxidative stress. We postulate that low doses of p-cresol induce mild oxidative stress that stimulates BDNF release by activating redox-sensitive genes [14]. Thus, BDNF secreted via the TrkB receptor could alter NF remodeling and modulate synaptic plasticity. Interestingly, one speculation of the present study is that cell death and cell survival-promoting mechanisms are altered by the intensity of oxidative stress, which is regulated by various p-cresol concentrations. Considering that the primary source of endogenous p-cresol is the intestinal microbiome, its positive or pathogenic effect depends on the composition and type in the gut. Thus, the balance between gut microbiome strains (especially Clostridium species) may directly influence neuroplasticity.

In the present study, we also revealed that oxytocin could modify p-cresol-dependent BDNF synthesis, in addition to opioidergic compounds. Oxytocin in dopaminergic neurons has been shown to attenuate ROS formation, which appears to be initiated by dopamine oxidation [26]. In addition, our previous studies have shown that oxytocin can inhibit the p-cresol-dependent elevation of the NR2B subunit of NMDA glutamate receptors in the rat nucleus accumbens [10]. Collectively, these data suggest that the p-cresol-dependent formation of BDNF could be modified by oxytocin in the dopaminergic structures of the brain. Nevertheless, elucidating the underlying mechanisms of these interactions requires further experimental confirmation.

It was previously reported that opioid transmitters increase NFs and BDNF, thus improving brain plasticity [27]. Increasing brain plasticity is an important process that contributes to learning, memory, and communication skills [28]. It is also known that with age, although the number of nerve cells in the brain remains unaltered, the plasticity of the brain decreases [29]. Therefore, in the present study, we aimed to determine the simultaneous action of a brain plasticity reducer (p-cresol) and opioid transmitters on nerve cells to clarify the underlying mechanism in a model simulating the precise condition.

In addition, it is known that *C. deficile*, considered the primary producer of p-cresol in the human body, became the dominant bacterium in the human ancestral microbiota 7 million years ago and, to date, remains predominant [30]. This means that the human microbiota produces p-cresol in a stable mode from the inception of hominid evolution. Researchers assessing p-cresol extensively discussed the possibility of a microbial imbalance that causes the release of substantial amounts of p-cresol and, consequently, induces neurodevelopmental complications. However, the precise role of p-cresol remains needs to be clarified when circulating in small quantities, i.e., in a “safe” subtoxic dose present in the blood and brain without neurological complications.
